# Identification of health-related quality of life profiles among long-term survivors of primary central nervous system tumors

**DOI:** 10.1007/s11060-023-04474-5

**Published:** 2023-10-30

**Authors:** Macy L. Stockdill, Tito Mendoza, Terri S. Armstrong, Christine Miaskowski, Bruce Cooper, Elizabeth Vera

**Affiliations:** 1 Neuro-Oncology Branch, National Cancer Institute, National Institutes of Health, Bethesda, USA; 2 School of Nursing, University of California San Francisco, San Francisco, USA

**Keywords:** Cancer, Functional status, Health-related quality of life, Survivors

## Abstract

**Purpose:**

We aimed to identify health-related quality of life (HRQOL) latent classes among primary central nervous system tumor (PCNST) long-term survivors (LTS) and to evaluate differences between classes in survivor sociodemographic characteristics, clinical characteristics, and symptoms to guide  the development of survivorship care programs tailored to unique class needs.

**Methods:**

Data from 298 PCNST LTS reporting HRQOL on the EQ-5D-3L were analyzed using latent profile analysis. Correlations and independent group *t*-tests were performed to identify differences between identified HRQOL classes by sociodemographic, clinical characteristics, and symptoms.

**Results:**

Sample mean age was 48 years, 54% were male, 82% Caucasian, 56% employed, 60% had a high-grade glioma, and 52% had a KPS ≥ 90. Two HRQOL classes, good (61%) and poor (39%), were identified. The good HRQOL class reported no problems with self-care and few problems with mobility or usual activities. Thirty-eight percent reported anxiety and depression and 21% pain. Over 94% of the poor HRQOL class had at least moderate problems with mobility and usual activities, and over 50% had pain, self-care issues, anxiety, and depression. Older age (*φ* = 0.21), unemployment (*φ* = 0.30), spine tumors (*φ* = 0.18), active treatment (*φ* = 0.20), tumor recurrence (*φ* = 0.28), and poorer KPS scores (*φ* = 0.61) were associated with membership in the poor HRQOL class.

**Conclusions:**

In the poor PCNST LTS HRQOL class, an overwhelming majority faced significant physical challenges, and the good HRQOL class experienced mood-related disturbance but limited physical challenges. These HRQOL profiles can be used to guide survivorship programs and tailored interventions.

**Supplementary Information:**

The online version contains supplementary material available at 10.1007/s11060-023-04474-5.

## Introduction

 Primary central nervous system tumors (PCNSTs) are rare with an average annual age-adjusted incidence rate of 24.7 per 100,000 [1], but their diagnosis is devastating for survivors. Primary central nervous system tumor survivors, including those with either a primary brain or spine tumor, report significant distress, an average of 10 concurrent symptoms, and functional limitations [2–4]. While prognosis is better among those with benign tumors who have an average 5-year relative survival rate of 91.8% compared to a rate of 35.7% among those with malignant tumors [1], both groups report continued cognitive complaints [5–8] and higher levels of anxiety and depression than the general population [4, 9]. Previous studies among young adult survivors of childhood onset PCNSTs report on the late effects of their disease and its treatment, including ongoing issues with neurocognitive functioning and health related quality of life (HRQOL) impairments [10]. Little is currently known about the overall HRQOL of adult survivors, as much of the research has focused on evaluation of the experience of adult survivors of childhood onset tumors [10, 11] or on the identification of factors associated with survival [12–15].

In these studies, the definition of a long-term survivor (LTS) varies, with LTS being identified as those living 2, 3, or 5 years or more after diagnosis [16, 17]. Previously, our research group conducted one of the first studies to explore prevalent survivorship symptom profiles among LTS ≥ 5 years from the time of diagnosis with low- and high-grade tumors and with tumors in both the brain and spine [17]. We identified that 42% of brain tumor LTS and nearly half of spine tumor LTS struggled with moderate to severe symptoms. Seventy-two percent of spine tumor LTS experienced activity-related symptom interference [17]. In the LTS adult brain tumor sample, LTS with more severe symptoms were more likely to be younger, unemployed, and have worse functional status [17]. Spine tumor LTS with severe symptoms were more likely to have worse functional status and have received previous treatment [17].

To continue to address the gap in knowledge on adult PCNST LTS HRQOL, we aimed to build on the results from our earlier report and identify latent classes of PCNST LTS with distinct HRQOL profiles and to evaluate the differences in sociodemographic and clinical characteristics and symptoms between profiles. Finally, we evaluated which sociodemographic and clinical characteristics were associated with individual dimensions of the HRQOL measure, the EQ-5D-3L. While evaluated sociodemographic and clinical characteristics were chosen based on previous literature, we used latent profile analysis to agnostically identify latent HRQOL profiles among a broad group of PCNST survivors, with the ultimate goal of developing this knowledge to guide future clinical survivorship programs for PCNST LTS.

## Materials and methods

### Study design

A cohort of PCNST LTS, defined as individuals living ≥ 5 years after a diagnosis of a PCNST, were identified from the National Cancer Institute Neuro-Oncology Branch’s Natural History Study (NOB-NHS; NCT #: NCT02851706; PI: Terri S. Armstrong). Five years was chosen for the definition of a LTS  based on previous work and as a way to incorporate individuals with both low- and high-grade tumors who are facing issues within the long-term survivorship phase [17]. The NOB-NHS is a longitudinal, observational study that follows individuals diagnosed with a PCNST along their disease trajectory. The NOB-NHS includes data on clinical outcomes and patient reported outcome questionnaires. Institutional Review Board approval was obtained for the NOB-NHS, and participants provided written informed consent.

#### Outcome measures

Data were collected during clinical evaluation of NOB-NHS LTS between September 2016 and December 2021. Participants were enrolled in the NOB-NHS across the phases of their disease trajectory.

#### Sociodemographic and clinical information

We collected information on LTS sociodemographic and clinical characteristics including age, sex, race, ethnicity, employment status, education level, income, months from diagnosis, tumor location, tumor type, tumor grade, tumor recurrence, active treatment status, and history of radiation. Functional status was assessed using the Karnofsky Performance Scale (KPS). Karnofsky Performance Scale scores range from 0 to 100 with higher scores indicating an individual is better able to carry out daily activities [18]. A KPS cut off of 80 has been demonstrated to identify differences between patient groups in relation to symptom burden and interference [19]. Therefore, for this analysis we dichotomized KPS scores as either good (≥ 90) or poor (≤ 80).

#### Patient reported outcomes measures

Self reported HRQOL was assessed using the EuroQol 5 Dimension 3 Levels (EQ-5D-3L) [20]. The EQ-5D-3L is comprised of five dimensions (i.e., mobility, self-care, usual activities, pain/discomfort, and anxiety/depression). These dimensions can be reported as the number and frequency of participants experiencing problems related to each dimension. Each dimension is scored from 1 to 3 (1 = no problems; 2 = some problems; 3 = extreme problems). The EQ-5D-3L is broadly used in studies among the PCNST population [17, 21].

Symptoms were measured using the MD Anderson Symptom Inventory-Brain Tumor module (MDASI-BT) or the MD Anderson Symptom Inventory-Spine Tumor module (MDASI-SP). The MDASI-BT and MDASI-SP assess symptom severity on a scale of 0 (“not present”) to 10 (“as bad as you can imagine”) [22]. The MDASI-BT subscales include an overall symptom severity score and six symptom factors, as well as a mean overall interference score comprised of activity and mood-related interference [22]. The MDASI-SP subscales include an overall symptom severity score and four symptom factors as well as an overall interference score comprised of activity and mood-related interference [22]. Both MDASI modules are well validated and reliable [19, 22, 23].

Anxiety and depressive symptoms were assessed using the Patient Reported Measurement Information System Short Form (PROMIS SF) v1.0-Anxiety 8a and Depression 8a, respectively. The PROMIS Depression and Anxiety scales are summed scores where higher scores indicate higher anxiety and depression [24, 25]. Scores are converted to T-scores with population means equal to 50 and a standard deviation of 10 [25]. On the PROMIS SF, anxiety and depressive symptoms are considered to be moderate-to-severe at 1 standard deviation above the population mean (T-score > 60).

#### Statistical analysis

Latent profile analysis in Mplus V8.8 [26] was used to identify distinct HRQOL classes based on individual ordinal self-ratings of the five dimensions of the EQ-5D-3L. Latent profile analysis is a person-centered mixture model statistical approach used to identify latent subpopulations or classes of individuals in a population based on unobserved variables [27]. Estimation was carried out with Robust Maximum Likelihood using a logit link. Fit indices used to assess the optimal number of classes included the Bayesian Information Criterion, Vuong-Lo-Mendell-Rubin likelihood ratio test, and entropy. In addition, identified classes were evaluated for conceptual or clinical significance as determined by the research and clinical team. Classes were named based on the pattern discerned from the five dimensions of the EQ-5D-3L.

Descriptive statistics were calculated in IBM SPSS Statistics, Version 28 [28] to describe participant sociodemographic characteristics, clinical characteristics, and patient reported outcome measures. Associations with sample sociodemographic and clinical characteristics were identified with Phi correlation coefficients, point biserial correlations, and Cramer’s V with 95% confidence intervals. Differences between the HRQOL groups and patient reported outcomes were assessed with independent group *t*-tests, and Cohen’s d with 95% confidence intervals were reported as effect sizes. For the overall sample, the five EQ-5D-3L dimensions were dichotomized to either “no problems” or “some problems or extreme problems” to accommodate skewed distributions and obtain reliable estimates of the associations. Phi correlation coefficients and Cramer’s V with 95% confidence intervals were calculated to assess for differences among the overall sample based on EQ-5D-3L dimensions. Significance levels were adjusted for multiple comparisons with the Holm-Bonferroni’s correction, where appropriate.

## Results

### Sample characteristics

Table 1 presents the sociodemographic and clinical characteristics of the sample of PCNST LTS (n = 298) and each HRQOL class. Among the total sample, associations between patient characteristics and EQ-5D-3L dimensions are provided in Supplementary Table 1. Only being unemployed and poorer KPS scores were associated with LTS reporting some to extreme problems on all five EQ-5D-3L dimensions. Being on active treatment, having a prior recurrence or undergone radiation therapy, and a lower income level were associated with LTS reporting some to extreme problems on at least one EQ-5D-3L dimension.

**Table 1:** Differences in demographic and clinical characteristics between two latent classes

	Total sample N = 298		Good HRQOL class N = 183		Poor HRQOL class N = 115		Effect size^a^	Sig.
	N	%	N	%	N	%		
Age, *mean (SD)*	48.41 (12.97)		46.27 (12.58)		51.82 (12.90)		0.21^+++^ (0.10–0.31)	< 0.001*
Median; Range	48; 21–85		44; 21–77		52; 24–85			
Months from diagnosis, mean (SD)	144.18 (70.44)		138.30 (57.09)		153.56 (87.05)		0.11^+++^ (− 0.01–0.22)	0.097
Median; Range	128; 60–456		132; 61–424		127; 60–456			
Sex								
Female	137	46	85	46	52	45	0.01^+^ (− 0.10–0.13)	0.836
Male	161	54	98	54	63	55		
Race								
White	244	82	148	81	96	83	− 0.01^+^ (− 0.13–0.11)	0.861
Other^b^	37	13	23	13	14	12		
Ethnicity^c^								
Non-Hispanic or Latino	268	90	162	89	106	92	− 0.08^+^ (− 0.19–0.04)	0.211
Hispanic or Latino	17	6	13	7	4	3		
Employment								
Employed	168	56	125	68	43	37	0.30^+^ (0.19–0.40)	< 0.001*
Unemployed	122	41	55	30	67	58		
Education								
≤ High School	31	10	22	12	9	8	0.06^++^ (− 0.06–0.17)	0.540
Some college or technical degree	150	50	93	51	57	50		
≥ Advanced degree	105	35	63	34	42	37		
Income								
< $50,000	34	11	18	10	16	14	0.01^++^ (− 0.17–0.19)	0.664
$50,000–$149,999	62	21	37	20	25	22		
≥ $150,000	24	8	12	7	12	10		
Location								
Brain	267	90	172	94	95	83	0.18^+^ (0.07–0.29)	0.002*
Spine	31	10	11	6	20	17		
Tumor grade								
Low grade (I/II)	106	36	70	38	36	31	0.03^++^ (− 0.08–0.14)	0.138
High grade (III/IV)	178	60	102	56	76	66		
Other	14	4	11	6	3	3		
Tumor classifications								
Glioblastoma	45	15	29	16	16	14		
Oligodendro-glial tumors	59	20	37	21	22	20		
Ependymoma	48	16	21	11	27	23		
Active treatment								
No	248	83	163	89	85	74	0.20^+^ (0.09–0.30)	< 0.001*
Yes	50	17	20	11	30	26		
Received radiation								
No	51	17	38	21	13	11	0.12^+^ (0.01–0.23)	0.035
Yes	247	83	145	79	102	89		
Prior recurrence								
No	90	30	74	40	16	14	0.28^+^ (0.17–0.38)	< 0.001*
Yes	208	70	109	60	99	86		
KPS								
≥ 90	157	52	135	74	22	19	0.61^+^ (0.53–0.68)	< 0.001*
≤ 80	111	37	28	15	83	72		

*HRQOL *health related quality of life, *KPS* Karnofsky performance status

*Denotes significance. Bonferroni-holm corrections were used to adjust for 14 levels of testing

^a^Effect sizes calculated using measures of association and their 95% Confidence Intervals (Phi^+^ for 2 × 2 tables, Cramer’s V^++^ for tables larger than 2 × 2 and point biserial correlation^+++^ between a binary variable and continuous variable)

^b^*The Other racial group consisted of those who identified as Asian (n = 14), Black or African American (n = 20), Native American or Pacific Islander (n = 1), and as Other than the groups listed (n = 2)

^c^Of the 298 participants reporting, 13 reported their Ethnicity was unknown and were not included in analysis.

### Latent classes for long-term survivor health-related quality of life

Latent profile analysis identified two classes: good (n = 183) and poor HRQOL (n = 115). Differences in sociodemographic and clinical characteristics between the two latent classes are presented in Table 1. Fit indices and details for the 2-class model status are presented in Table 2. Membership in the poor HRQOL class was associated with being older (*φ* = 0.21, CI [0.10–0.31], *p* < 0.001), unemployed (*φ* = 0.30, CI [0.19–0.40], *p* < 0.001), having a spine tumor (*φ* = 0.18, CI [0.07–0.29], *p* = 0.002), being on active treatment (*φ* = 0.20, CI [0.09–0.30], *p* < 0.001), having a prior recurrence (*φ* = 0.28, CI [0.17–0.38], *p* < 0.001), and a lower KPS score (*φ* = 0.61, CI [0.53–0.68], *p* < 0.001).

**Table 2:** Good and poor HRQOL latent profile solutions and fit indices for 1 through 3 classes

Model	LL	AIC	BIC	Entropy	VLMR
1 Class	− 1177.52	2375.03	2412.00	n/a	n/a
2 Class^a^	− 981.78	2005.57	2083.21	0.90	391.47^†^
3 Class	− 958.75	1981.49	2099.80	0.94	46.08*

*AIC* Akaike’s information criterion, *BIC* Bayesian information criterion, *HRQOL* health related quality of life, *LL* log-likelihood, *n/a* not applicable, *ns* not significant, *VLMR* Vuong-Lo-Mendell-Rubin likelihood ratio test for the K vs. K-1 model

*p < 0.05; ^†^p < 0.00005

^a^The 2-class solution was selected because the BIC for that solution was lower than the BIC for the 1-class (baseline) solution. In addition, the VLMR was significant for the 2-class solution, indicating that two classes fit the data better than one class. Although the VLMR was significant for the 3-class solution, the BIC for the 3-class solution was larger than the BIC for the 2-class solution, indicating that too many classes had been extracted. Further, the 3-class solution included a small predicted class (only 6 predicted cases; 2-percent of the sample), raising the concern that the solution is unlikely to generalize to other samples

### Health related quality of life dimensions

Table 3 summarizes the HRQOL dimensions of the overall sample and the HRQOL classes. The poor HRQOL class was characterized by over 95% having at least some issues with mobility and usual activities and over 57% having problems with self-care, anxiety and depression, and pain. While most patients in the good HRQOL class reported no to little problems with mobility, usual activities, or self-care issues, 38% reported some problems with anxiety and depression and 21% pain. The two HRQOL classes differed on all EQ-5D-3L dimensions with the poor HRQOL reporting more problems on all five EQ-5D-3L dimensions.

**Table 3:** HRQOL class distribution for each of the EQ-5D-3L dimensions for the overall sample and latent classes

	Overall sample N = 298		Good HRQOL class N = 183		Poor HRQOL class N = 115		Effect size^a^	Sig.
	N	%	n	%	n	%		
Mobility								
No problems walking about	173	58	167	91	6	5	0.85 (0.81–0.88)	< 0.001*
Some or extreme problems walking about	125	42	16	9	109	95		
Self-care								
No problems with self-care	232	78	183	100	49	43	0.67 (0.61–0.73)	< 0.001*
Some or extreme problems washing or dressing myself	66	22	0	0	66	57		
Usual activities								
No problems with usual activities	159	53	158	86	1	0	0.83 (0.80–0.87)	< 0.001*
Some or extreme problems with usual activities	139	47	25	14	114	100		
Pain/discomfort								
No pain or discomfort	184	62	145	79	39	34	0.45 (0.36–0.54)	< 0.001*
Moderate or extreme pain or discomfort	114	38	38	21	76	66		
Anxiety/depression								
Not anxious or depressed	151	51	113	62	38	33	0.28 (0.17–0.38)	< 0.001*
Moderately or extremely anxious or depressed	147	49	70	38	77	67		

*EQ-5D-3L* EuroQol 5 dimension 3 levels, *HRQOL* health related quality of life

*Denotes significance. Bonferroni-holm corrections were used to adjust for 5 levels of testing for the 5 EQ-5D-3 L dimensions.

^a^Effect size measured by Phi correlation and 95% Confidence Intervals

### Association of health-related quality of life class with self-reported symptom burden, interference, and mood

Among those with brain tumors, the poor HRQOL class had higher severity of symptoms overall, higher scores on the 6 symptom factor groupings, and higher scores for symptom interference on the MDASI-BT. Table 4 presents the differences in PROMIS, MDASI-BT, and MDASI-SP scores between the two latent classes. The poor HRQOL class had a significantly higher MDASI-BT overall symptom burden scores (M = 2.8, SD = 1.7) compared to the good HRQOL class (M = 1.3, SD = 1.5) (t(263) = − 7.29, *p* < 0.001, Cohen’s *d* = - 0.93), and a higher overall interference score (M = 4.6, SD = 2.6) compared to the good HRQOL class (M = 1.3, SD = 1.8) (t(144) = − 10.95, p < 0.001, Cohen’s *d* = - 1.56). Among spine tumor LTS, the poor HRQOL class had a significantly higher overall symptom burden score (M = 3.1, SD = 1.9) on the MDASI-SP compared to the good HRQOL class (M = 1.2, SD = 1.4) (t(27) = − 2.77, *p* = 0.01, Cohen’s *d* = - 1.08), and also had a higher overall interference score (M = 5.1, SD = 2.7) compared to the good HRQOL class (M = 1.2, SD = 2.3), (t(27) = − 3.95, *p* = 0.001, Cohen’s *d* = - 1.54). The poor HRQOL class (M = 4.7, SD = 2.3) reported worse scores on the disease-related factor score (weakness, numbness, pain, sleep disturbance, and fatigue) of the MDASI-SP when compared to the good HRQOL class (M = 1.4, SD=1.9) (t(27) = − 3.86, *p* < 0.001, Cohen’s *d* = - 1.51). The poor HRQOL class had a higher depressive t-score (M = 55.1, SD = 9.6) and a higher anxiety t-score (M = 53.9, SD = 10) compared to the good HRQOL class depression (M = 47.3, SD = 8.8) (t(296) = − 7.25, *p* < 0.001, Cohen’s *d* = - 0.86), and anxiety scores (M = 48.9, SD = 9.6) (t(296) = − 4.32, *p* < 0.001, Cohen’s *d* = - 0.51). The class differences based on overall symptom burden scores and symptom interference scores for both brain and spine tumor LTS, as well as differences in the two classes on depression scores, demonstrated large effect sizes while anxiety scores between the groups demonstrated a medium effect size.

**Table 4:** Differences in PROMIS, MDASI-BT, and MDASI-SP between the two latent classes

	Good HRQOL class N = 183			Poor HRQOL classN = 115			Effect size^a^	Sig.
	n	Mean	SD	N	Mean	SD		
MDASI-BT								
Overall symptom burden	170	1.3	1.5	95	2.8	1.7	-0.93 (− 1.20–-0.67)	< 0.001*
Affective symptom factor	170	2.0	2.2	95	3.8	2.6	-0.79 (− 1.05–-0.53)	< 0.001*
Cognitive symptom factor	170	1.7	2.2	95	3.4	2.7	-0.70 (− 0.96–-0.44)	< 0.001*
Neurologic symptom factor	170	0.9	1.6	95	2.6	2.0	-0.94 (− 1.21–00.68)	< 0.001*
Treatment related symptom factor	170	1.2	1.7	95	2.6	2.0	-0.78 (− 1.03–0.52)	< 0.001*
General disease symptom factor	170	0.8	1.3	95	2.0	1.9	-0.77 (− 1.03–-0.51)	< 0.001*
GI symptom factor	170	0.5	1.2	95	0.9	1.7	-0.33 (− 0.59–-0.08)	0.018*
Overall interference	170	1.3	1.8	95	4.6	2.6	-1.56 (− 1.84–-1.27)	< 0.001*
Activity related interference	170	1.3	1.9	95	5.1	2.8	-1.69 (− 1.98–-1.40)	< 0.001*
Mood related interference	170	1.3	1.9	95	4.1	2.9	-1.22 (− 1.49–-0.95)	< 0.001*
MDASI-SP								
Overall symptom burden	10	1.2	1.4	19	3.1	1.9	-1.08 (− 1.89–-0.25)	0.01*
Disease related symptom factor	10	1.4	1.9	19	4.7	2.3	-1.51 (− 2.36–-0.63)	< 0.001*
Autonomic function symptom factor	10	0.8	1.1	19	2.7	3.0	-0.74 (− 1.52–0.06)	0.023
Constitutional/treatment symptom factor	10	0.9	1.4	19	1.4	1.6	-0.29 (− 1.05–0.48)	0.467
Emotional symptom factor	10	1.8	2.2	19	3.1	3.3	-0.45 (− 1.22–0.33)	0.260
Overall interference	10	1.2	2.3	19	5.1	2.7	-1.54 (− 2.40–-0.66)	0.001*
Activity related interference	10	1.3	2.4	19	5.7	2.6	-1.76 (− 2.64–-0.85)	< 0.001*
Mood related interference	10	1.1	2.3	19	4.5	3.0	-1.23 (− 2.06–-0.39)	0.004*
PROMIS-depression								
T-score	183	47.3	8.8	115	55.1	9.6	-0.86 (− 1.11–-0.62)	< 0.001*
Depressive severity^b^								
* Moderate-severe (n, %)*	18	10		33	29			
PROMIS-anxiety								
T-score	183	48.9	9.6	115	53.9	10.0	-0.51 (− 0.75–-0.28)	< 0.001*
Anxiety severity^b^								
* Moderate-severe (n, %)*	23	13		25	22			

*KPS* Karnofsky performance status, *HRQOL* health related quality of life, *MDASI-BT* MD Anderson symptom inventory-brain tumor module, *MDASI-SP* MD Anderson symptom inventory-spine tumor module, *PROMIS-Depression* patient reported measurement information system short form v1.0-depression, *PROMIS-Anxiety* patient reported measurement information system short form v1.0-anxiety

*Denotes significance. Bonferroni-holm corrections were used to adjust for 7 levels of testing for MDASI-BT symptom factor scales, 3 levels for the MDASI-BT symptom interference scales, 5 levels for the MDASI-SP symptom burden scale, and 3 levels for the MDASI-SP interference scale

^a^Effect sizes calculated using Cohen’s d and their 95% Confidence Intervals (for independent *t*-tests)

^b^PROMIS SF anxiety and depressive symptoms are considered to be moderate-to-severe at 1 standard deviation abobe the population mean (T-score > 60)

## Discussion

This study is the first to use latent profile analysis to identify HRQOL classes of PCNST LTS. In our sample, unemployment and worse functional status was associated with LTS membership in the poor HRQOL class. This study supports that in PCNST survivorship, the functional limitations and burdensome symptoms of a survivor’s tumor may be key determinants of HRQOL that we should consider. Survivorship care needs may be very different based on these determinants, instead of solely the grade of tumor. A decline in functional status, which is a known prognostic factor across many tumors, including among individuals with a glioblastoma [29], can interfere with many daily activities, including employment. Comparable to other reports among glioma and meningioma populations, 40% of our sample was unemployed 5 years after diagnosis [29–31]. Disease related functional limitations that prevent PCNST LTS from working may contribute to the ongoing anxiety and depression reported by LTS [4, 32], including glioma LTS [29], and vocational rehabilitation should be considered in survivorship programs for these individuals.

Long-term survivors in the poor HRQOL class were more likely to experience tumor recurrence or be on active treatment, suggesting more aggressive disease, despite longer-term survival. Aggressive tumor types may be related to worse functioning and wellbeing for individuals with a PCNST [33], and treatment associated symptoms may have contributed to their worse HRQOL.  Eighty-three percent of our sample had received radiation therapy, but radiation was not associated with belonging in the poor HRQOL class. However, radiation and tumor resection following recurrence are associated with declines in cognitive function among many PCNST survivors [34] which ultimately may affect HRQOL. Previous work with a subsample of this cohort revealed that radiation therapy was related to worse overall symptoms and symptom interference [17], which mirrors reports of the negative effects of radiation on worse quality of life among PCNST survivors [35]. Active MRI surveillance is also necessary for PCNST survivors to monitor for disease progression, and scanxiety in patients undergoing active treatment for recurrence may contribute to heightened mood disturbance including anxiety, distress, and fear of cancer recurrence or death [36, 37]. In Neuro-Oncology survivorship care, we often don’t use words like ‘remission’ and patients, regardless of tumor grade, often continue with surveillance imaging for the remainder of the survivors’ lives. This study found that those who had had a tumor recurrence during their course of their disease and more treatment had worse HRQOL indicating that the impact of treatment on how the patient feels and functions deserves focused research and understanding longitudinally to reduce this effect [38]. This also highlights the need for ongoing support of emotional needs and screening for depression and anxiety even into survivorship for these individuals.

While the number of spine tumor LTS in our sample was small (n = 31), 64% belonged to the poor HRQOL class. Approximately 66% of those in the poor HRQOL class reported issues with pain/discomfort. In supplementary analysis, 75% of the thirty-one spine tumor LTS reported pain or discomfort. Spine tumor LTS-across tumor grades and without active disease-continue to report pain [17, 39]. Individuals with a spine tumor commonly report symptoms such as pain, fatigue, numbness, weakness in extremities, and more activity-related interference compared to their counterparts with a brain tumor, with reports indicating a high percentage require narcotic analgesia to manage pain long term [17, 23, 40]. Spine tumor LTS who perceive their symptoms as interfering with functional status may experience worse mood-related symptoms and report worse HRQOL, highlighting the importance of effective pain management in survivorship programs for these individuals.

Long-term survivors in the poor HRQOL class had higher overall symptom burden and interference scores on the MDASI measures and higher PROMIS depression and anxiety scores. The good HRQOL class reported almost no issues with mobility, self-care or usual activity abilities, but 21% reported moderate to severe pain and 38% reported anxiety or depression. Routine symptom assessment in patients with other cancers have shown that patients are not only more likely to have less depression and improved HRQOL with routine assessment, but also have less emergency room visits and live longer [41]. Previous reports by our group have identified that both patients with tumors in the brain [3] and spine [17, 39] experience more than 10 concurrent symptoms. Routine assessment of anticipated symptoms based on disease location and treatment, evidence-based strategies for management, and evaluation of interventions are needed in survivorship programs in both groups. Symptom assessment should include anxiety and depression as LTS in both classes deal with everyday stressors, stresses of ongoing MRI surveillance, recurrence fears, and existential distress [42]. These strategies as well as those needed to manage pain may be different between the classes, with preserving function and independence as the priority strategy to address pain and anxiety or depression among those with impaired functional status.

### Limitations

Because this study was cross-sectional, we cannot infer causality and cannot state if our sample had health effects from the time of their diagnosis or if these health effects were cumulative or late effects of either their tumor or treatment. Participants had to be able to self-report to be included in this analysis, and there may have been patients with more significant health or cognition issues in the general PCNST LTS population that declined to participate in this study. In addition, the sample of spine tumor LTS was small, which limited our ability to detect group differences. However, spine tumors are rare and underrepresented in current PCNST research and may be a small part of some Neuro-Oncology practices. Therefore, including spine tumor LTS is an important component of our study to provide guidance on survivorship care for these individuals and is a strength of our study. The sensitivity of our analysis and our ability to identify additional HRQOL classes may be limited by our sample size which may not be generalizable to other populations, age ranges, or other regions, but represents one of the largest reports of PCNST LTS published to date. Our sample was also limited in that it was mostly White/Caucasian and included participants who sought out care at the NCI, which may have led to bias in our results.

## Conclusion

Future research should focus on collecting longitudinal data from PCNST LTS, including spine tumor LTS, to better understand the trajectory of HRQOL in LTS and develop interventions to meet their needs. Further identification of the longitudinal contributing factors to PCNST survivors’ HRQOL will aid in the development of either prevention or management strategies. Furthermore, while several supportive interventions exist for individuals diagnosed with a PCNST, interventions need to be developed to support the growing population of long-term PCNST survivors along their illness trajectory. Care plans and supportive interventions targeting PCNST LTS with better HRQOL may fare better with an approach focused on helping PCNST LTS to cope with their chronic condition and on-going surveillance, maintaining function, and adjusting back to life and daily activities. Interventions for PCNST LTS with worse HRQOL facing significant physical limitations may require more intensive rehabilitative based approaches to facilitate fostering independence. Informal caregivers will be fundamental to provide support to LTS throughout this process and should be included in care planning and intervention planning and evaluation. It is important to continue to assess physical health and function related to the HRQOL of LTS as patients dealing with tumor recurrence, ongoing treatments, and spine tumors may face significant challenges. Maintaining functional status among these group of individuals may be even more important. The HRQOL profiles we have identified can be used to develop broad survivorship care plans and supportive care interventions across tumor types for those PCNST LTS dealing with similar issues.

### Supplementary Information

Below is the link to the electronic supplementary material.

Supplementary material 1 (DOCX 35.9 kb)
