# Supplementary material for: Identification of health-related quality of life profiles among long-term survivors of primary central nervous system tumors
Source: J Neurooncol. 2023 Oct 30;165(1):181–90. doi: 10.1007/s11060-023-04474-5 (PMC10638191; doi:10.1007/s11060-023-04474-5)
Supplement: Supplementary file 1 — Supplementary material 1 (DOCX 35.9 kb) [file 11060_2023_4474_MOESM1_ESM.docx]

**Full Title:** Identification of Health-related Quality of Life Profiles Among Long-term Survivors of Primary Central Nervous System Tumors

**Journal:** Journal of Neuro-Oncology

**Authors:** Macy L. Stockdill^1^ (MLS), Tito Mendoza^1^ (TM), Terri S. Armstrong^1^ (TSA), Christine Miaskowski^2^ (CM), Bruce Cooper^2^ (BC), Elizabeth Vera^1^ (EV)

**Affiliations:** ^1^Neuro-Oncology Branch, National Cancer Institute, National Institutes of Health, U.S.A

^2^School of Nursing, University of California San Francisco, U.S.A

**Correspondence:** Macy L. Stockdill, PhD, BSN

Neuro-Oncology Branch, National Cancer Institute, National Institutes of Health

9030 Old Georgetown Road, Room 233

Bethesda, MD, 20892

*Email:* macy.stockdill@nih.gov

| ***Supplemental Table 1.* Differences in EQ-5D-3L Dimensions Based on Overall Sample Characteristics** | | | | | | | | | | | | |  | | | |  | | | |
| --- | --- | --- | --- | --- | --- | --- | --- | --- | --- | --- | --- | --- | --- | --- | --- | --- | --- | --- | --- | --- |
| **EQ-5D-3L** | **Mobility** | | | | **Self-Care** | | | | **Usual Activities** | | | | **Pain/Discomfort** | | | | **Anxiety/Depression** | | | |
|  | NP* | SP/EP* | Effect Size^a^ | Sig^b^ | NP | SP/EP | Effect Size | Sig. | NP | SP/EP | Effect Size | Sig. | NP | SP/EP | Effect Size | Sig. | NP | SP/EP | Effect Size | Sig. |
| **Sex** |  |  |  |  |  |  |  |  |  |  |  |  |  |  |  |  |  |  |  |  |
| *Female* | 76 | 61 | -0.05^+^  (-0.16-0.07) | 0.405 | 107 | 30 | 0.01^+^  (-0.11-0.12) | 0.924 | 76 | 61 | 0.04^+^  (-0.07-0.15) | 0.499 | 85 | 52 | 0.01^+^  (-0.11-0.12) | 0.922 | 69 | 68 | -0.01^+^  (-0.12-0.11) | 0.922 |
| *Male* | 97 | 64 |  |  | 125 | 36 |  |  | 83 | 78 |  |  | 99 | 62 |  |  | 82 | 79 |  |  |
| **Race** |  |  |  |  |  |  |  |  |  |  |  |  |  |  |  |  |  |  |  |  |
| *White* | 142 | 102 | .03^+^  (-0.09-0.14) | 0.635 | 188 | 56 | -0.01^+^  (-0.13-0.11) | 0.857 | 130 | 114 | 0.03^+^  (-0.09-0.15) | 0.599 | 155 | 89 | 0.08^+^  (-0.03-0.20) | 0.155 | 122 | 122 | -0.08^+^ (-0.20-0.04) | 0.168 |
| *Non-white* | 20 | 17 |  |  | 29 | 8 |  |  | 18 | 19 |  |  | 19 | 18 |  |  | 23 | 14 |  |  |
| **Ethnicity** |  |  |  |  |  |  |  |  |  |  |  |  |  |  |  |  |  |  |  |  |
| *Non-Hispanic* | 153 | 115 | -0.06^+^  (-0.18-0.05) | 0.274 | 205 | 63 | -0.10^+^  (-0.21-0.02) | 0.132 | 139 | 129 | -0.09^+^  (-0.20-0.03) | 0.134 | 164 | 104 | -0.05^+^  (-0.16-0.07) | 0.439 | 142 | 126 | 0.08^+^  (-0.03-0.20) | 0.157 |
| *Hispanic* | 12 | 5 |  |  | 16 | 1 |  |  | 12 | 5 |  |  | 12 | 5 |  |  | 6 | 11 |  |  |
| **Employment** |  |  |  |  |  |  |  |  |  |  |  |  |  |  |  |  |  |  |  |  |
| *Employed* | 120 | 48 | 0.31^+^  (0.20-0.41) | <0.001* | 149 | 19 | 0.30^+^  (0.19-0.40) | <0.001* | 109 | 59 | 0.26^+^  (0.15-0.37) | <0.001* | 115 | 53 | 0.18^+^  (0.06-0.29) | 0.002* | 97 | 71 | 0.17^+^  (0.05-0.28) | 0.005* |
| *Unemployed* | 50 | 72 |  |  | 78 | 44 |  |  | 47 | 75 |  |  | 62 | 60 |  |  | 50 | 72 |  |  |
| **Education** |  |  |  |  |  |  |  |  |  |  |  |  |  |  |  |  |  |  |  |  |
| *High School* | 19 | 12 | 0.04^++^  (-0.08-0.15) | 0.831 | 28 | 3 | 0.06^++^  (-0.06-0.17) | 0.259 | 18 | 13 | 0.04^++^  (-0.07-0.16) | 0.775 | 16 | 15 | -0.10^++^  (-0.22-0.01) | 0.215 | 18 | 13 | -0.04^++^  (-0.08-0.16) | 0.699 |
| *College Degree* | 89 | 61 |  |  | 116 | 34 |  |  | 82 | 68 |  |  | 90 | 60 |  |  | 76 | 74 |  |  |
| *Advanced Degree* | 59 | 46 |  |  | 82 | 23 |  |  | 54 | 51 |  |  | 71 | 34 |  |  | 52 | 53 |  |  |
| **Income** |  |  |  |  |  |  |  |  |  |  |  |  |  |  |  |  |  |  |  |  |
| *<$50,000* | 18 | 16 | -0.01^++^  (-0.19-0.17) | 0.875 | 25 | 9 | -0.06^++^  (-0.23-0.12) | 0.602 | 15 | 19 | -0.04^++^  (-0.22-0.14) | 0.845 | 12 | 22 | -0.34^++^  (-0.49--0.17) | <0.001* | 13 | 21 | -0.16^++^  (-0.33-0.02) | 0.187 |
| *$50,000-$149,999* | 36 | 26 |  |  | 51 | 11 |  |  | 31 | 31 |  |  | 39 | 23 |  |  | 29 | 33 |  |  |
| *>$150,000* | 13 | 11 |  |  | 19 | 5 |  |  | 12 | 12 |  |  | 20 | 4 |  |  | 15 | 9 |  |  |
| **Tumor Location** |  |  |  |  |  |  |  |  |  |  |  |  |  |  |  |  |  |  |  |  |
| *Brain* | 162 | 105 | 0.16^+^  (0.04-0.26) | 0.007* | 213 | 54 | 0.14^+^  (0.02-0.25) | 0.019 | 148 | 119 | 0.12^+^  (0.01-0.23) | 0.035 | 177 | 90 | 0.27^+^  (0.17-0.38) | <0.001* | 136 | 131 | 0.02^+^  (-0.10-0.13) | 0.788 |
| *Spine* | 11 | 20 |  |  | 19 | 12 |  |  | 11 | 20 |  |  | 7 | 24 |  |  | 15 | 16 |  |  |
| **Tumor Grade** |  |  |  |  |  |  |  |  |  |  |  |  |  |  |  |  |  |  |  |  |
| *Low grade* | 69 | 37 | 0.06^++^ (-0.06-0.17) | 0.03 | 87 | 19 | 0.05^++^  (-0.06-0.16) | 0.271 | 62 | 44 | 0.01^++^  (-0.10-0.12) | 0.01 | 57 | 49 | -0.13^++^  (-0.24--0.02) | 0.067 | 54 | 52 | 0.05^++^  (-0.06-0.17) | 0.077 |
| *High grade* | 93 | 85 |  |  | 133 | 45 |  |  | 85 | 93 |  |  | 116 | 62 |  |  | 94 | 84 |  |  |
| *Other* | 11 | 3 |  |  | 12 | 2 |  |  | 12 | 12 |  |  | 11 | 3 |  |  | 3 | 11 |  |  |
| **Active Treatment** |  |  |  |  |  |  |  |  |  |  |  |  |  |  |  |  |  |  |  |  |
| *No* | 154 | 94 | 0.18^+^  (0.07-0.29) | 0.002* | 198 | 50 | 0.11^+^  (-0.01-0.22) | 0.066 | 141 | 107 | 0.16^+^  (0.04-0.27) | 0.007 | 157 | 91 | 0.07^+^  (-0.04-0.18) | 0.217 | 123 | 125 | -0.05^+^  (-0.16-0.07) | 0.409 |
| *Yes* | 19 | 31 |  |  | 34 | 16 |  |  | 18 | 32 |  |  | 27 | 23 |  |  | 28 | 22 |  |  |
| **Received Radiation** |  |  |  |  |  |  |  |  |  |  |  |  |  |  |  |  |  |  |  |  |
| *No* | 40 | 11 | 0.19^+^  (0.08-0.30) | 0.001* | 46 | 5 | 0.14^+^  (0.02-0.25) | 0.02 | 35 | 16 | 0.14^+^  (0.03-0.25) | 0.02 | 35 | 149 | 0.06^+^  (-0.05-0.18) | 0.27 | 22 | 29 | -0.07^+^  (-0.18-0.05) | 0.24 |
| *Yes* | 133 | 114 |  |  | 186 | 61 |  |  | 124 | 123 |  |  | 16 | 98 |  |  | 129 | 118 |  |  |
| **Prior Recurrence** |  |  |  |  |  |  |  |  |  |  |  |  |  |  |  |  |  |  |  |  |
| *No* | 73 | 17 | 0.31^+^  (0.20-0.41) | <0.001* | 82 | 8 | 0.21^+^  (0.10-0.32) | <0.001* | 69 | 21 | 0.31^+^  (0.20-0.41) | <0.001* | 60 | 30 | 0.07^+^  (-0.05-0.18) | 0.25 | 48 | 90 | 0.04^+^  (-0.08-0.15) | 0.545 |
| *Yes* | 100 | 108 |  |  | 150 | 58 |  |  | 90 | 118 |  |  | 124 | 84 |  |  | 103 | 105 |  |  |
| **KPS** |  |  |  |  |  |  |  |  |  |  |  |  |  |  |  |  |  |  |  |  |
| *>90* | 130 | 27 | 0.62^+^  (0.54-0.69) | <0.001* | 150 | 7 | 0.51^+^  (0.42-0.59) | <0.001* | 123 | 34 | 0.58^+^  (0.49-0.65) | <0.001* | 114 | 43 | 0.30^+^  (0.18-0.40) | <0.001* | 96 | 61 | 0.20^+^  (0.09-0.32) | <0.001* |
| *<80* | 23 | 88 |  |  | 58 | 53 |  |  | 22 | 89 |  |  | 48 | 63 |  |  | 45 | 66 |  |  |
| ^a^Effect sizes calculated using measures of association and their 95% Confidence Intervals (Phi^+^ for 2x2 tables, Cramer’s V^++^ for tables larger than 2X2 and point biserial correlation^+++^ between a binary variable and continuous variable).  ^b^*The Other racial group consisted of those who identified as Asian (n=14), Black or African American (n=20), Native American or Pacific Islander (n=1), and as Other than the groups listed (n=2).  ^c^Of the 298 participants reporting, 13 reported their Ethnicity was unknown and were not included in analysis.  *Denotes significance. Bonferroni-holm corrections were used to adjust for 13 levels of testing  Abbreviations: NP = No Problems; SP/EP = Some Problems/Extreme Problems | | | | | | | | | | | | | | | | | | | | |
